# Photodynamic killing of cancer cells by a Platinum(II) complex with cyclometallating ligand

**DOI:** 10.1038/srep22668

**Published:** 2016-03-04

**Authors:** Rachel E. Doherty, Igor V. Sazanovich, Luke K. McKenzie, Alexander S. Stasheuski, Rachel Coyle, Elizabeth Baggaley, Sarah Bottomley, Julia A. Weinstein, Helen E. Bryant

**Affiliations:** 1 Academic Unit of Molecular Oncology, Department of Oncology, Sheffield Cancer Research Centre, University of Sheffield, Beech Hill Road, Sheffield S10 2RX, United Kingdom; 2 Department of Chemistry, University of Sheffield, Sheffield S3 7HF, United Kingdom; 3 Institute of Physics, Minsk, Belarus

## Abstract

Photodynamic therapy that uses photosensitizers which only become toxic upon light-irradiation provides a strong alternative to conventional cancer treatment due to its ability to selectively target tumour material without affecting healthy tissue. Transition metal complexes are highly promising PDT agents due to intense visible light absorption, yet the majority are toxic even without light. This study introduces a small, photostable, charge-neutral platinum-based compound, Pt(II) 2,6-dipyrido-4-methyl-benzenechloride, complex **1**, as a photosensitizer, which works under *visible* light. Activation of the new photosensitizer at low concentrations (0.1–1 μM) by comparatively low dose of 405 nm light (3.6 J cm^−2^) causes significant cell death of cervical, colorectal and bladder cancer cell lines, and, importantly, a cisplatin resistant cell line EJ-R. The photo-index of the complex is 8. We demonstrate that complex **1** induces irreversible DNA single strand breaks following irradiation, and that oxygen is essential for the photoinduced action. Neither light, nor compound alone led to cell death. The key advantages of the new drug include a remarkably fast accumulation time (diffusion-controlled, minutes), and photostability. This study demonstrates a highly promising new agent for photodynamic therapy, and attracts attention to photostable metal complexes as viable alternatives to conventional chemotherapeutics, such as cisplatin.

Over 10.9 million people are diagnosed with cancer worldwide every year^1^. The current treatment options for patients include surgery, chemotherapy and radiotherapy. However surgical intervention is not always possible and existing chemotherapies, whilst highly effective in many cases, especially with targeted delivery, still often cause high levels of non-cancerous cell death and associated toxicity. Whilst enormous progress has been made in this area, less invasive and more tumour specific therapies are in continuous need.

A long known and currently re-emerging concept for cancer treatment is the use of light-activated chemotherapeutics, a process termed photodynamic therapy (PDT)^2^,^3^,^4^,^5^,^6^.

PDT is based on the use of compounds which are only toxic to cells following exposure to a specific wavelength of light. The necessity for light activation allows the tumour mass to be specifically targeted whilst the drug remains inert elsewhere in healthy tissue which has not been exposed to light. This approach significantly reduces toxicity in healthy tissues^7^,^8^.

Photosensitizers are broadly classified into three groups, according to their mechanism of action. In all cases, the primary act is absorption of light by the photosensitizer (PS), populating its singlet excited state, ^1^PS*. Type I and type II are two classes of mechanism of ROS/singlet oxygen generation, in both, PDT is initiated by an interaction of the photosensitizer in its excited state with oxygen, with the production of reactive oxygen species (ROS) including superoxide anion (O_2_^•–^), hydroxyl radical (OH•), or hydrogen peroxide (H_2_O_2_), in type I, or singlet oxygen (type II), which then induce the damage leading to cell death^9^,^10^. In the third class of mechanism of photo-activated cell death, which can be referred to as Photo-activated Chemotherapy (PACT), killing is initiated by a direct reaction of the photosensitizer in the excited state (or of products of its light-induced transformation) with biomolecules, leading to cell death that is independent of oxygen^11^.

Oxygen dependent mechanisms rely on the interaction of the excited state of the sensitizer with oxygen. Therefore, the compounds with longer-lived excited states are required to maximize the yield of the primary step in the therapy. Transition metal complexes are particularly promising in this regard for two main reasons. Firstly, this class of compounds usually absorb visible (and in some cases NIR) light strongly. Secondly, the singlet excited (S_1_) populated upon absorption of light (S_0_ → S_1_) efficiently populates the triplet excited state (T_1_), via intersystem crossing, due to strong spin-orbit coupling promoted by the heavy metal atom^12^. Because of spin-forbidden nature of T_1_ → S_0_ transition, T_1_ triplet excited states are significantly longer-lived (microseconds) than singlet excited states of organic compounds (S_1_ → S_0_), which typically decay within a few nanoseconds.

Recently, several examples of transition metal complexes as either oxygen dependent or independent photosensitizers in PDT have been reported, including Ru(II), Re(I) and Pt(IV)^9^,^13^,^14^,^15^,^16^,^17^,^18^,^19^,^20^,^21^,^22^,^23^,^24^,^25^,^26^,^27^,^28^. Here, we demonstrate the potential of a small, low-weight Pt(II) complex for PDT. We have previously reported Pt(II)-based compounds of dipyridobenzene and derivatives, which intensely absorb light in UV/visible region, possess triplet excited state with remarkably long (microseconds) lifetime^29^,^30^, and produce singlet oxygen in 70–80% yield in organic solvents such as dichloromethane, these reports included complex **1** (previously abbreviated as [PtL^3^Cl]). Furthermore, the compounds are readily taken up by mammalian cells, predominantly residing in the nuclear compartment. Such a favorable combination of properties prompted our investigation of this class of compounds as potential PDT agents.

The present study demonstrates the light-specific cell toxicity of a representative Pt(II) compound bearing cyclometallating dipyrido-benzene type ligand - complex **1** (Fig. 1A) in a range of cancer cells including a cisplatin resistant line, and investigates its interaction with DNA and the type of DNA damage induced in cancer cells by this new photosensitizer. We note that complex **1** was abbreviated [PtL^3^Cl] in our earlier reports^29^,^30^.

## Results

### Photodynamic killing of cancer cells by complex 1

**Figure 1:**
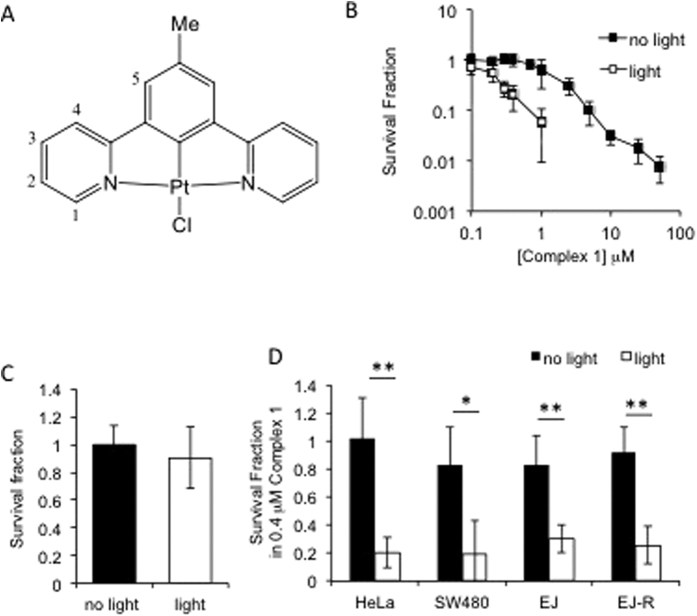
Photodynamic killing of cancer cells by complex **1**. (**A**) Structure of complex **1**. (**B**) Survival fractions of HeLa cells following exposure to increasing doses of complex **1**. Cells were pre-treated with complex **1** prior to 3 min exposure to 405 nm light (3.6 J cm^−2^). (**C**) Survival fractions of HeLa cells exposed to light in the absence of complex **1**. (**D**) Survival fractions of cervical cancer (HeLa), colorectal cancer (SW480), bladder cancer (EJ) and cisplatin-resistant bladder cancer (EJ-R) cells. Cells were pre-treated with 400 nM complex **1** prior to 3 min exposure to 405 nm light. Data shown are mean and standard deviation of at least 3 independent experiments. Significance was determined using the Student’s T-test where n = 3 and p < 0.01 is indicated by ^**^, and p < 0.05 is indicated by ^*^. Survival fractions were calculated relative to untreated control.

We have previously described the use of complex **1** (Fig. 1A) as an emission imaging agent, where experiments were performed over the course of hours following a 5 min incubation time. Within the 24 h window required for imaging, the toxicity established by MTT assay was negligible^29^. In the present study investigations of long term dark and light-induced toxicity have been undertaken. complex **1** was added to cells, followed by exposure to 405 nm light (3.6 J cm^−2^) and cellular viability examined at several time-points up to 12 days post exposure. Figure 1B demonstrates the cytotoxic effect of complex **1** with and without exposure to light in the human cancer cell line HeLa. The data demonstrate a significant increase in the toxicity of complex **1** following exposure to light at a complex **1** concentration as low as 0.1 μM. Furthermore, complex **1** without exposure to light remains non-toxic to cells up to a dose of 1 μM. LD50 values in HeLa cells are 0.2 μM in light and 1.6 μM in dark, making the photo-index of this complex 8. There was no significant cell death following exposure to light alone (Fig. 1C).

Similar light-induced toxicity of complex **1** with low-level toxicity in the dark has been observed in two further cancer cell lines, the colorectal cancer cell line SW480 and the bladder cancer cell line EJ (Fig. 1D): in each case exposure to light caused complex **1**-induced cell death. Moreover, light-induced complex **1**-mediated cell death occurred in the cisplatin-resistant bladder cancer cell line EJ-R (Fig. 1D), thus suggesting that it could be an alternative therapy for cancers which are resistant to current platinum-based drugs. The light specific cell death demonstrated here and the low dark toxicity of complex **1** highlights its potential as an appropriate PDT agent.

### Complex 1 binding to DNA

**Figure 2:**
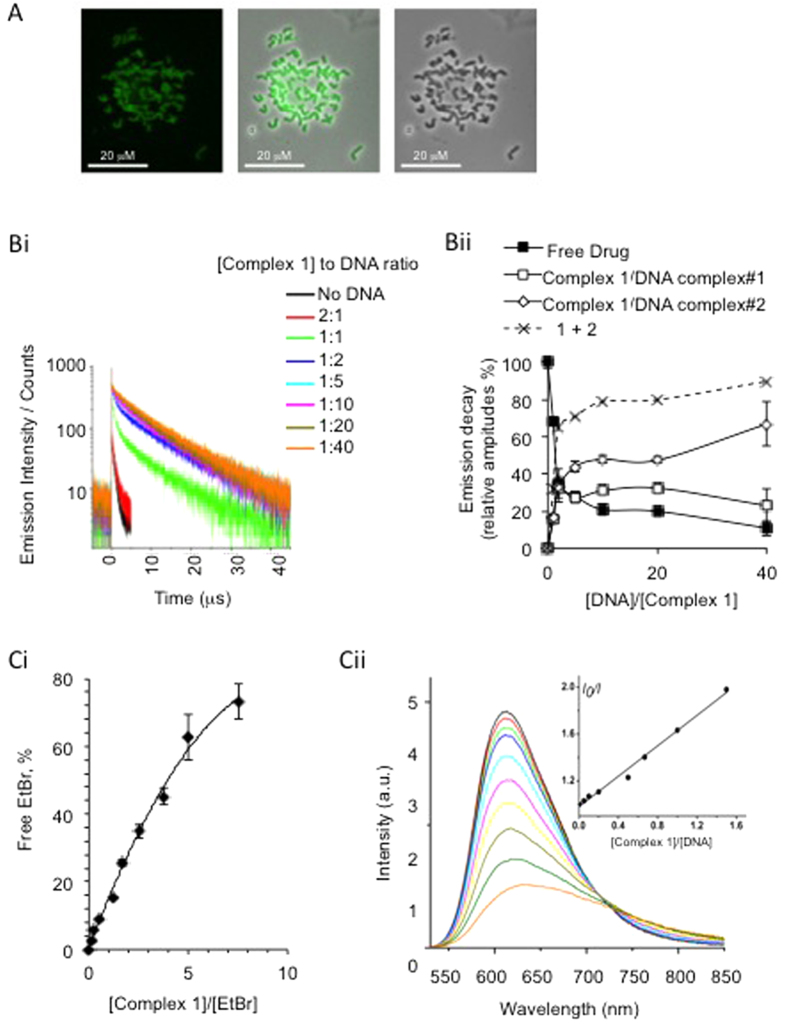
Complex **1** binding to DNA. (**A**) Metaphase spread of chromosomes extracted from HeLa cells that had been pre-incubated with complex **1**. Emission image (left); light microscope image (right) and merged image (centre). (**Bi)** Emission decay of complex **1** recorded at 525 – 575 nm range as a function of concentrations of calf thymus DNA under 410 nm pulsed excitation. (**Bii)** Relative amplitudes of the emission decay component attributed to the unbound -▪-, intercalated -□-, and partially intercalated -⋄- [complex **1**] at different DNA concentrations. –x- is a sum of the relative contribution of the two DNA-bound forms, -⋄- and -□-. (**C)** Competitive DNA binding of complex **1** versus EtBr.25 μM calf thymus DNA was incubated with 10 μM EtBr, followed by titration with complex **1** solution. The emission spectrum of the displaced EtBr has been measured. (**Ci**) Increase in relative emission intensity of free EtBr upon addition of [complex **1**] to the DNA pretreated with EtBr. (**Cii**) Emission spectra from which the data in Ci were obtained. The inset shows the plot used to determine the binding constant, see text for details.

Complex **1** has been shown to accumulate within the nucleus of cells^29^ and the planar coordination, polycyclic structure and aromaticity of the compound suggest that some degree of DNA intercalation is likely. We therefore investigated the ability of complex **1** to bind DNA in cells. Cells were incubated with complex **1** for 30 min, chromosome metaphase spreads were prepared and imaged using multiphoton excitation (λex = 780 nm) (Fig. 2A). Chromosomes were stained with complex **1** indicating that nuclear localization is due at least in part to accumulation in DNA. The mode of DNA binding was then examined. In the presence of calf thymus DNA in solution, the emission decay of complex **1** can be described as biexponential, with the lifetimes of 3 (±0.5) μs, and 11(±0.5) μs (Fig. 2B). The 11 μs value is close to the radiative lifetime of the excited state of complex **1**^29^, and indicates some protection from oxygen, most likely though intercalation^31^,^32^. The presence of a 3 μs decay component, which is still considerably longer than the anticipated lifetime for complex **1** emission (<1 μs) in an air-equilibrated media, suggests that an additional mode of binding exists, most likely groove binding. We speculate these binding modes may allow some limited access of oxygen to complex **1** which would cause partial quenching of the triplet excited state.

To further support the hypothesis that complex **1** interacts with DNA we demonstrated its ability to competitively bind to DNA versus the known DNA intercalator Ethidium Bromide (EtBr). Figure 2C shows that increasing the concentration of complex **1** correlates with an increase in displacement of EtBr bound to DNA into solution, therefore demonstrating complex **1** is able to intercalate into DNA in the same manner as EtBr. Assuming a binding constant of 1.0 × 10^7^ M^−1^ for EtBr^33^, the binding constant determined for complex **1** by this method is 1.19 (±0.08) 10^5^ M^−1^. Significantly adduct formation with G-N7 position of DNA was tested and found to be negative (supporting information S4).

### Light-activated complex **1** induces DNA single strand breaks (nicks) in plasmid DNA

**Figure 3:**
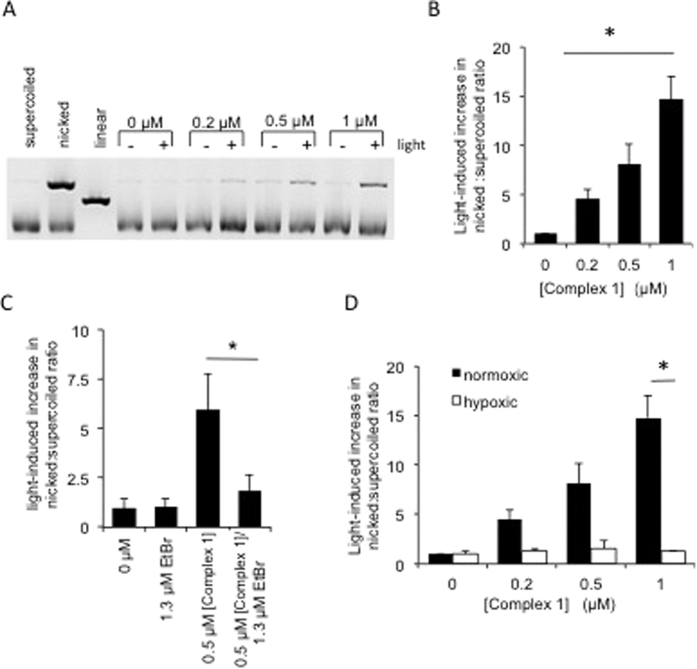
Light-induced activation of complex **1** induces DNA single strand breaks (DNA nicks) in plasmid DNA and is inhibited by addition of ethidium bromide or in hypoxic conditions. (**A)** Electrophoresis of PUC19 plasmid DNA treated with (+) and without (−) 405 nm light (3.6 J cm^−2^) and between 0 μM and 1 μM of complex **1**. 0.3 μg DNA was loaded from each sample. Lanes 1–3 represent control DNAs where nicks/single strand breaks were induced by rapid freeze thawing of DNA and linearization induced using a restriction endonuclease. (**B)** Quantification of the light-induced change in ratio of nicked to supercoiled DNA. Values represent fold increase in ratios in light exposed compared to non-exposed samples. (**C)** Quantification of the light-induced change in ratio of nicked to supercoiled DNA when ethidium bromide (EtBr) was included in the reaction. (**D)** Quantification of the light-induced change in ratio of nicked to supercoiled DNA when reactions were carried out under normoxic or under hypoxic (0.1% oxygen) conditions. Data shown are mean and standard deviation of at least 3 independent experiments. Significance was determined using the Student’s T-test where n = 3 and p < 0.05 is indicated by ^*^.

Having established light-induced cell killing by complex **1**, the next step was to determine whether complex **1** induces DNA damage, and if so whether such damage is light dependent. Therefore plasmid DNA was incubated with increasing concentrations of complex **1**, followed by irradiation with 405 nm light; a parallel experiment without irradiation was performed in each case. The presence of single strand breaks (SSBs) in DNA was determined by agarose gel electrophoresis (Fig. 3). Circular plasmid DNA which has had a SSB introduced loses its supercoiled shape and therefore is unable to migrate as far through an agarose gel as more compact supercoiled DNA, such DNA is often termed “nicked” DNA. In this way, the presence of a second band at a higher weight in an agarose gel indicates the presence of nicked DNA i.e. SSBs in a sample (control lane 2). Figure 3 clearly demonstrates that complex **1** induces DNA SSBs only after light activation. Neither complex **1** nor light alone were sufficient to induce DNA SSBs. Furthermore the DNA nicking effect of light-activated complex **1** was concentration dependent, with the largest increase in nicked DNA being present at the highest concentration of 1 μM (Fig. 3B). The absence of induction of the linear form of plasmid of DNA (seen as an intermediate migrating band - linear control lane 3), suggests that light-activated complex **1** does not induce double strand breaks in DNA.

Addition of EtBr into the reaction reduced induction of SSBs in plasmid DNA (Fig. 3C), suggesting that *in vitro* light-activated DNA damage by complex **1** is dependent upon a mode of complex **1**-DNA binding which is inhibited by the presence of EtBr. In addition performing the reaction in the absence of oxygen (hypoxic conditions) prevented induction of SSBs (Fig. 3D) demonstrating that complex **1**-induced breaks are dependent upon oxygen. Taken together these data suggest that breaks are induced when light activated complex **1** is bound to DNA in a binding mode in which complex **1** has access to oxygen.

### Light-activated complex **1** induces single strand DNA breaks in cancer cells

**Figure 4:**
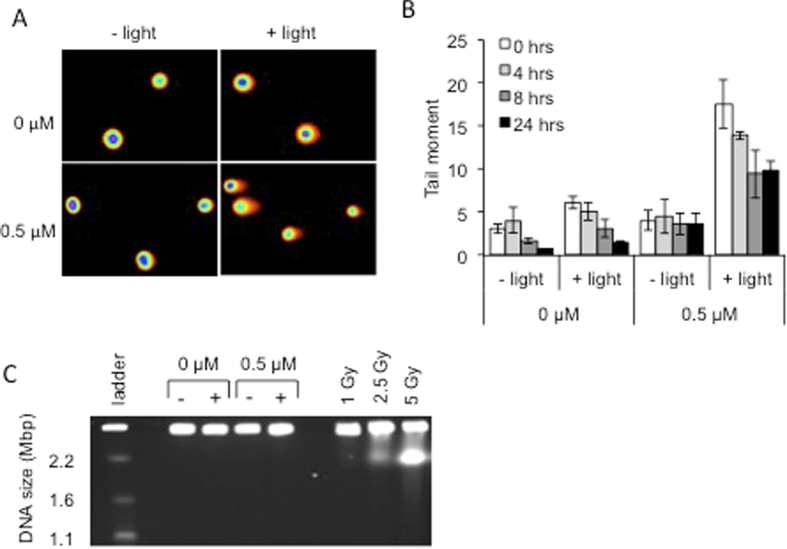
Light-induced complex **1** activity selectively induces DNA single strand breaks within cells. (**A)** Representative COMET assay images for each of the treatments. Images were obtained using the full spectrum function of the CometScore™ computer software. Cells were originally stained with SYBR Safe™ DNA gel stain. (**B)** Average Tail Moment (TM) in HeLa cells at various time points following treatment with 0.5 μM complex **1** with and without exposure to 405 nm light (3.6 J cm^−2^). The average TM was calculated using CometScore™ software, where at least 50 cells were analysed on each of 3 occasions and the standard deviation is shown. (**C)** Pulsed-field gel electrophoresis to visualise DSBs in cells treated with 0.5 μM complex **1** with and without exposure to 405 nm light. Cells were harvested immediately after treatment. Gamma irradiation (1 – 5 Gy) of cells was used as positive control for the induction of DSBs.

In order to determine whether complex **1** is also capable of inducing DNA damage within cells upon irradiation with light, a neutral COMET assay was used. This assay enables the detection of both DSBs and SSBs in DNA within individual cells^34^, by lysing cells and then performing single cell electrophoresis - any damaged DNA migrates out of the cell as a ‘tail’ (Fig. 4A). Four treatment groups were set up, DMSO control, complex **1** alone, DMSO with light, and complex **1** with light. Cells from each group were harvested for analysis of DNA breaks at several time points up to 24 h post treatment. The results show a large number of DNA breaks induced immediately following complex **1** and light treatment (Fig. 4B), the majority of this damage was not repaired 24 h post treatment. Significantly lower levels of DNA damage were observed in cells treated with complex **1** or light alone.

The COMET assay is not able to distinguish between DSBs and SSBs in DNA and therefore pulsed field gel electrophoresis (PFGE) was performed on cells immediately following complex **1** and light treatment (Fig. 4C). PFGE only detects DSBs in DNA^35^, it is not able to detect SSBs, in this way we were able to determine whether the breaks observed in the COMET assay were either SSBs or DSBs. Gamma irradiated cells were used as positive control for DSBs in DNA. Figure 4C clearly demonstrates the absence of DSBs in treated cells, confirming the DNA breaks observed in treated cells using the COMET assay protocol are indeed SSBs and not DSBs. This result supports the observations from plasmid DNA that SSBs not DSBs are induced by light-activated complex **1**. As unrepaired DNA strand breaks are known to cause cell death^36^, these data strongly suggest that the light-induced complex **1**-mediated specific killing of cancer cells observed in Fig. 1 is caused by the induction of DNA breaks which are unable to be repaired in cells.

## Discussion and Conclusions

The results presented above can be summarized as follows. In several cancer cell models complex **1** has been demonstrated to behave as a promising photosensitizer under visible light (405 nm light, light dose 3.6 J cm^−2^). The typical light dose range used in PDT with small metal complexes is ~2–30 J cm^−2^, demonstrating that complex **1**-induced photosensitizion can be caused by comparatively low doses of light^13^,^16^,^21^,^22^,^23^,^24^,^26^. The LD50 values for complex **1** in HeLa cells are 0.2 μM with light and 1.6 μM without, giving a photo-index of 8. It is worth noting that while other compounds have been shown to have values of up to 3 magnitudes higher^22^,^23^, many other small metal complexes are reported with similar photo-indexes of <10^16^,^17^,^21^,^26^.

Both light or compound alone induced a low level of DNA strand breaks which are quickly repaired in cells. However in the presence of light and complex **1** a large number of DNA strand breaks are induced; these breaks are not fully repaired by 24 h post induction, a time frame under which one would expect to see repair^37^. In circular DNA in solution, addition of EtBr as well as using hypoxic conditions prevented DNA break induction, indicating that breaks are dependent both on complex **1**-DNA binding and on the presence of oxygen for their formation. This suggests that breaks are the result of ROS/singlet oxygen creation upon light mediated activation of DNA bound complex **1**.

In apparent contradiction to these data, while complex **1** photosensitizes singlet oxygen efficiently (70%)^29^ in organic solvents, in the presence of DNA in H_2_O, no singlet oxygen was detected within 5% limit of the equipment used (see^36^ for method description), which is consistent with the majority of the complex being fully protected from oxygen. However these *in vitro* data do not exclude the possibility that in cells a portion of DNA bound complex **1** can generate sufficient singlet oxygen or other ROS in the presence of light to induce DNA SSBs and cause cell killing.

It has been stated above that complex **1** interacts with the DNA via at least two different mechanisms. An emission lifetime of 11 μs (which is close to the radiative lifetime for this compound at room temperature), together with the substitution studies using EtBr indicate one of the modes of complex **1**–DNA interaction is likely to be intercalation or at least partial intercalation. This mode of DNA interaction, is documented for other transition metal complexes with planar structures similar to complex **1**^38^. Intercalation is expected to give complete protection from oxygen, while partial intercalation would allow for creation of ROS/singlet oxygen molecules upon light mediated activation of complex **1**. An additional different mechanism of binding of complex **1** to DNA is indicated by the emission lifetime component of up to 3 μS. This value is less than the radiative lifetime, but larger than the emission lifetime observed in an air-saturated solution of complex **1**, and is indicative of a partial protection from oxygen. We speculate that this effect is due to external binding, perhaps to the minor groove of DNA, which could also allow for creation of ROS/singlet oxygen molecules upon light mediated activation of complex **1**. Thus while data with circular DNA demonstrates that DNA binding and oxygen are both required for induction of DNA SSBs and data generated in complex **1** treated cells suggest that it is irreversible DNA SSBs that are responsible for killing cells, it is not clear which of the two modes of DNA binding detected in solution is occurring in cells and is responsible for DNA SSBs or cell killing.

Several routes of photosensitization action of complex **1** can be speculated upon. Firstly, it is possible that in the presence of oxygen light itself induces damage to DNA which under usual circumstances are repaired through the existing cellular mechanisms. However, in the presence of complex **1**, these mechanisms are compromised, leading to irreversible damage. Secondly, it is possible that complex **1** engages in photoinduced electron transfer with one of the DNA bases, creating a radical-ion of the base, which then propagates the damage. Thirdly, it is possible that non-intercalated or partially-intercalated DNA bound complex **1** is not fully protected from oxygen, and is engaged either in the production of singlet oxygen (<5% yield may still be sufficient to initiate the process) or other ROS, which cause DNA single strand breaks. Each of these mechanisms assumes that it is damage of DNA that causes cell death, however it should also be remembered that complex **1** is localized throughout the nucleus and to a lesser extent the cytoplasm, therefore it is possible that non-DNA bound forms of the drug when activated by light are killing cells. The discussion of possible routes of action highlights the complexity of interpreting *in vitro* data when determining the mechanism of action of photosensitization in cells.

Although photosensitizers that target DNA could be argued to have the potential to induce mutations in cells, targeting of DNA for chemotherapy has long been an established and successful way of treating cancer. As with other agents, should this compound be taken forward to the clinic the potential for long-term side effects of therapy need to be weighed up against the increased survival benefit that could be achieved in patients.

Overall, it is clear that complex **1**, is a low-weight charge-neutral compound with high chemical- and photo-stability^29^. It is easy to prepare, rapidly penetrates cells, and can act as a photosensitizer in a diverse range of cancer cell lines including an example of cis-platin resistant cells. This highlights the potential of small light-absorbing metal complexes for PDT applications.

## Methods

### Compound

Complex **1**, Pt(2,6-dipyrido-4-Me-benzene)Cl, was prepared following the procedure described previously^30^ and characterized by NMR spectroscopy, elemental analysis, and high-resolution mass-spectroscopy. Analytical data (see Supplementary Figs S1–S3) are consistent with those reported previously. Detailed characterization of absorption and emission properties, singlet oxygen yield, and cellular uptake and localisation, have been reported by us previously^29^,^30^,^39^.

### Cell culture

The cancer cell lines used, HeLa (cervical), SW480 (colorectal) and EJ (bladder), were purchased from American Type Culture Collection. The EJ-R (cisplatin resistant) cell line was generated previously^40^. All cells were cultured in Dulbecco’s modified Eagles Medium (DMEM) with 10% fetal calf serum, penicillin (100 U ml^−1^) and streptomycin (100 μg ml^−1^) at 37 ^o^C in 5% CO_2_.

### Drug treatment

Complex **1** initially was dissolved in DMSO and stored at stock solutions of 0.05 mM, 0.5 mM and 5 mM at −20 °C, then further diluted in cell media for working concentrations which were kept upto 1 week at 4 ^o^C. Cells were trypsinised, washed with phosphate buffered saline (PBS) and re-suspended in phenol-red free DMEM in a soda glass vial. The re-suspended cells were treated with complex **1** solution, or with the same concentration of pure DMSO (vehicle control) and exposed to 405 nm light for 3 minutes (light source: M405L2 LED (Thorlabs, Newton, NJ, USA)) focused to 1 × 1 cm^2^ spot at sample, light power at sample adjusted to 20 mW), this equates to 3.6 J cm^−2^. Complex **1** concentration ranged from 50 nM to 100 μM. Control experiments without exposure to light were performed for each dosage to determine toxicity of DMSO and complex **1** in the absence of light. The maximum concentration of DMSO in biological experiments was 0.1%. The controls contained the same concentration as complex **1** treated samples.

### Toxicity assay

Between 2500 and 10,000 cells were treated as described above and subsequently plated onto 100 mm dishes. Following 10–12 days, when colonies could be observed, dishes were fixed and stained with 4 g/L methylene blue in methanol. Colonies consisting of more than 50 cells were then counted and the survival fractions for each dose compared to vehicle control cells were calculated.

### DNA cleavage assay

A total of 1.5 μg of PUC19 plasmid DNA was incubated in 50 mM Tris-HCl 18 mM NaCl, pH 7.2 buffer with either 0.5 μM complex **1** or DMSO in a total reaction mixture of 125 μl. Reaction mixtures were then exposed to light as described above. The control reactions without exposure to light for DMSO and 0.5 μM complex **1** were set up in parallel. All reactions were then incubated overnight at 37 °C. 5 μl of 0.05% bromophenol blue loading buffer was added to 25 μl of each reaction mixture prior to electrophoresis on a 1.2% agarose gel run at 100 V for 1 hour. Gels were stained with 1 μg/ml Ethidium Bromide (EtBr) and then visualised on a UGenius fluorescence gel imager (Syngene, Cambridge, UK). DNA bands were quantified using GelQuantNET software (Biochem lab solutions, San Francisco, Ca, USA). The ratio of nicked DNA versus supercoiled DNA was determined and then the fold change in that ratio between no light conditions and light conditions at each dosage was calculated. To determine DNA cleavage capacity of complex **1** following intercalation of EtBr, 19 μM of PUC19 plasmid DNA base pairs was saturated with 13.1 μM EtBr for 5 minutes prior to addition of 0.5 μM complex **1**. To determine DNA cleavage capacity of complex **1** under hypoxic conditions PUC19 plasmid DNA was incubated in an environment containing <0.1% oxygen in a hypoxic chamber (Don Whitley VA500 anaerobic workstation fitted with individually gassed humidified boxes (Don Whitley Scientific, Shipley, West Yorkshire) for 30 min in the dark prior to light treatment.

### Optical spectroscopy experiments

The emission spectra were recorded on Fluoromax 4 spectrofluorimeter (HORIBA Jobin Yvon). Emission lifetime measurements were performed on the mini-τ spectrofluorometer (Edinburgh Instruments), with 405 nm pulsed diode laser as an excitation source. The emission decays were detected in the spectral range 475–525 nm selected by a bandpass filter.

The competitive binding experiments for complex **1** vs. EtBr were performed as described previously^41^. The concentrations of calf thymus DNA were 25 μM DNA base pairs and 10 μM EtBr. The concentrations of added complex **1** were in the range of 0 to 75 μM. Calf thymus DNA (sodium salt, Type I, fibers) and EtBr (molecular biology grade) were purchased from Sigma Aldrich and used without further purification.

For each sample solution, the emission spectrum of EtBr was recorded under excitation at 513 nm. The emission spectrum of free EtBr in buffer without DNA at the same optical density as when bound to DNA was recorded and subtracted as background in the data processing. The integrated intensity of EtBr emission was plotted vs. concentration ratio of EtBr to complex **1** to obtain the binding constant for complex **1** intercalation into DNA.

To verify complex **1** intercalation into DNA, emission titrations of complex **1** solution (15 μM) with calf thymus DNA (in the range of 0 to 0.6 mM DNA base pairs) were performed, and the emission spectrum and emission decay of complex **1** were recorded as a function of DNA concentration (excitation at 380 nm for emission spectral measurements and at 405 nm for emission lifetime measurements). All the measurements were performed in quartz fluorimeter cuvettes (Starna Scientific, 1 cm pathlength). The UV-Vis absorption spectra were measured on Cary 50 Bio spectrophotometer to adjust sample concentrations and to confirm sample composition. All the samples were prepared by mixing appropriate amounts of the corresponding stock solutions. The concentrations of stock solutions of complex **1,** calf thymus DNA and EtBr were checked spectrophotometrically using the known extinction coefficients 6.78 × 10^3^ M^−1^ cm^−1^ at 412 nm for complex **1**^29^ 1.32 × 10^4^ M^−1^ cm^−1^ at 260 nm (per base pair) for calf thymus DNA^42^ and 5448 M^−1^ cm^−1^ at 480 nm for EtBr^43^. Tris-HCl buffer pH 7.4 was used in all emission experiments.

### COMET assay

COMET assay reagents were purchased from Trevigen and used according to the manufacturer’s neutral COMET protocol (Gaithersburg, MD, USA). Briefly, 1 × 10^6^ cells were either treated with DMSO or 0.5 μM complex **1** with and without light as described above. 2.5 × 10^5^ cells from each treatment were resuspended in 250 μl PBS. The remaining cells in each treatment were split into 3 separate plates and cultured in DMEM + 10% FCS to be analysed at 4 h, 8 h and 24 h post treatment. For COMET analysis 25 μl of cell suspension was mixed with 250 μl molten LMAgarose and then 75 μl of the mixture pipetted immediately onto COMET slides. LMAgarose was allowed to set at 4 °C for 20 minutes and then immersed in chilled lysis buffer for 30 minutes at 4 °C. Slides were then subjected to electrophoresis at 20 V for 45 minutes in chilled electrophoresis buffer (0.5 M Tris, 1.5 M sodium acetate). Following electrophoresis slides were incubated for 30 min in DNA precipitation solution (5 M ammonium acetate in 95% ethanol) at room temperature and then fixed in 70% ethanol for 20 min at room temperature. Slides were then allowed to dry and then each sample stained with 50 μl 1 × SYBR green before visualisation on a Nikon TE200 inverted fluorescent microscope (Nikon instruments, Kingston, UK). For each treatment the average tail moment of between 50 and 100 cells was calculated using the TriTek CometScore software (TriTek Corporation, Sumerduck, VA, USA).

### Pulse field gel electrophoresis (PFGE)

To identify double strand breaks (DSBs) in DNA 1 × 10^6^ cells were treated with either DMSO or 0.5 μM complex **1** with and without 405 nm light exposure, as described above. Cells were resuspended in 50 μl PBS and mixed with 50 μl InCert agarose (Lonza, Basel, Switzland). The mixture was then added to the plug insert and allowed to set at 4 °C for 20 minutes. Plugs were then incubated in 1 ml of 0.5 M EDTA, 1% N-laurylsarcosyl, proteinase K (1 mg/ml) buffer for 48 h at room temperature. Following incubation, plugs were washed 4 times in TE buffer and then loaded into a 0.7% chromosomal grade agarose gel. Separation by pulse field electrophoresis was subsequently performed for 24 h (Biorad; 120^o^ angle, 60–240 s switch time, 4 V/cm). After pulse field electrophoresis the gel was stained with EtBr and visualised on a UGenius fluorescence gel imager (Syngene, Cambridge, UK).

### Metaphase spread

2 × 10^6^ HeLa cells were incubated with 50 μM complex **1** for 10 min before colcemid (1:200 dilution of 10 ug/ml Gibco^®^ KaryoMAX^®^ Colcemid™) was added for 90 min. Cells were then trypsinised and washed. A solution of 0.075 M KCl and was added for 10 minutes. Cells were washed and fixative (3:1 methanol: acetic acid) was added. Cell solution in fixative was dropped onto moist, clean microscope slides and allowed to dry. A coverslip was added using mounting agent (Immu-Mount™, Thermo Scientific Shandon™). Slides were imaged using multiphoton excitation (λ_ex_ = 780 nm) on a Zeiss LSM 510 upright microscope.

## Additional Information

**How to cite this article**: Doherty, R. E. *et al*. Photodynamic killing of cancer cells by a Platinum(II) complex with cyclometallating ligand. *Sci. Rep.* **6**, 22668; doi: 10.1038/srep22668 (2016).

## Supplementary Material

Supplementary Information
