# Supplementary material for: Photodynamic killing of cancer cells by a Platinum(II) complex with cyclometallating ligand
Source: Sci Rep. 2016 Mar 4;6:22668. doi: 10.1038/srep22668 (PMC4778139; doi:10.1038/srep22668)
Supplement: Supplementary Information [file srep22668-s1.pdf]

Supplementary Information

Photodynamic killing of cancer cells by a Platinum(II) complex with cyclometallating ligand

Rachel E. Doherty[1], Igor V. Sazanovich[1,2,§], Luke K. McKenzie[1,2], Alexander S. Stasheuski,[3] Rachel Coyle[1], Elizabeth Baggaley[2], Sarah Bottomley[1], Julia A. Weinstein[2] and Helen E. Bryant[1]*

Characterisation of Complex 1

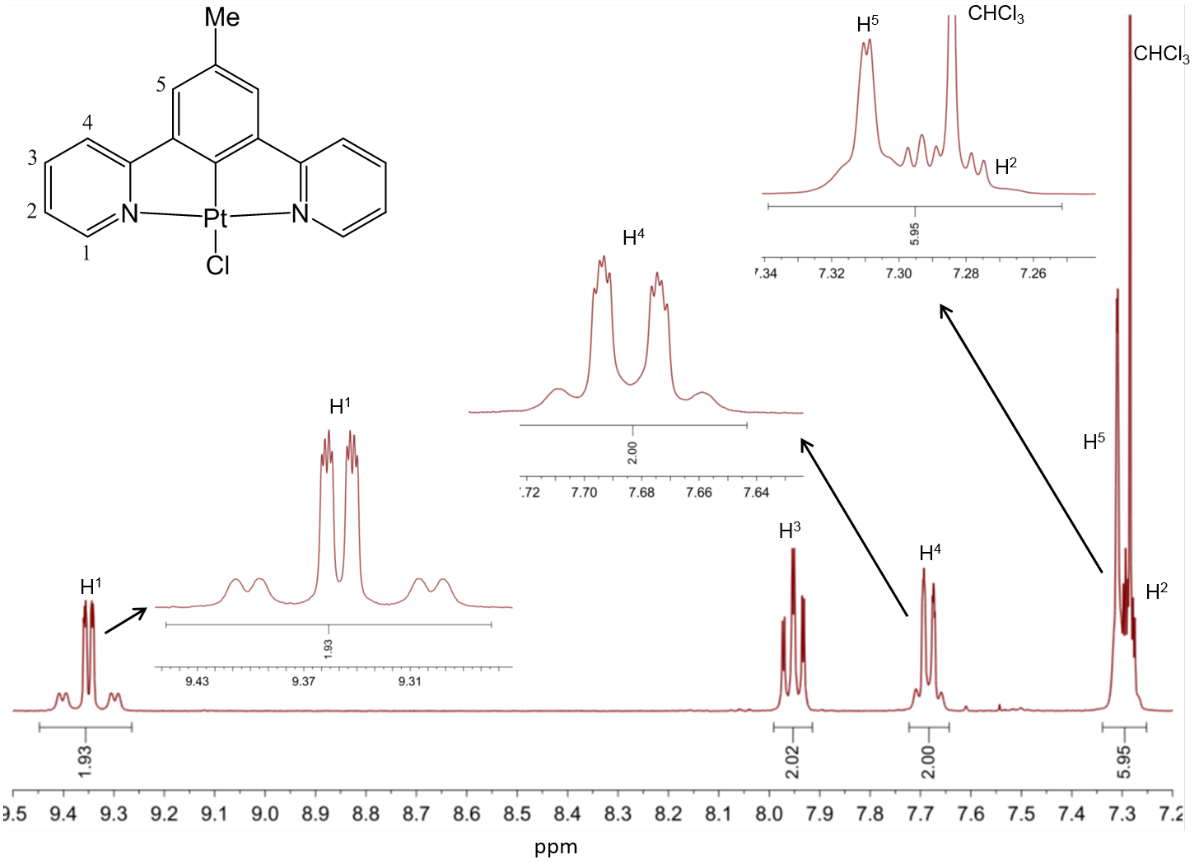

***Figure S1**: Molecular structure (top left) and ^{1}H NMR spectrum (CDCl3, 400 MHz) of Complex 1. Expansion of peaks included to show Pt satellites and splitting patterns.*

$^{13}C\{^1H\}$ NMR (400MHz, $CDCl_3$): δ_C = 22.1 (1C, s, Me), 119.1 (2C, s, *J* (^{13}C-^{195}Pt) 25 Hz, CH-Ar), 123.1 (2C, s, *J* (^{13}C-^{195}Pt) 16 Hz, CH-Ar), 124.9 (2C, s, *J* (^{13}C-^{195}Pt) 19, CH-Ar), 132.4 (2C, s C^{quat}), 139.0 (2C, s, CH-Ar), 140.8 (2C, s, C^{quat}), 152.2 (2C, s, CH-Ar) and 167.4 (1C, s, C^{quat}). Remaining quaternary carbon not detected.

Elemental Analysis. Calculated: C 42.91% H 2.75% N 5.89% Cl 7.45%; Found: C 42.64% H 2.63% N 5.73% Cl 7.54%.

Mass Spec. MS (EI): m/z = 476 $[MH]^+$, 440 $[M\text{-}Cl]^+$. MS $(ES)^+$: m/z = 440 $[M\text{-}Cl]^+$

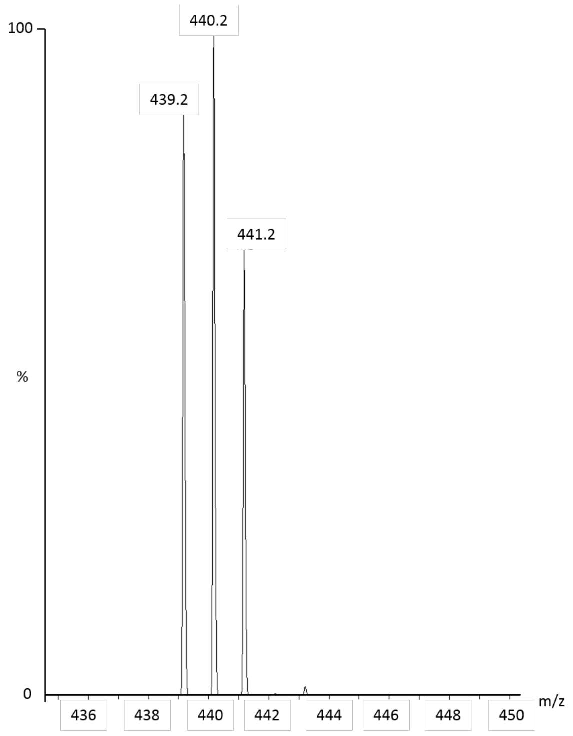

Figure S2: *ES mass spectrum of Complex 1 showing $[M\text{-}Cl]^+$ m/z= 440 peak with Pt isotope pattern.*

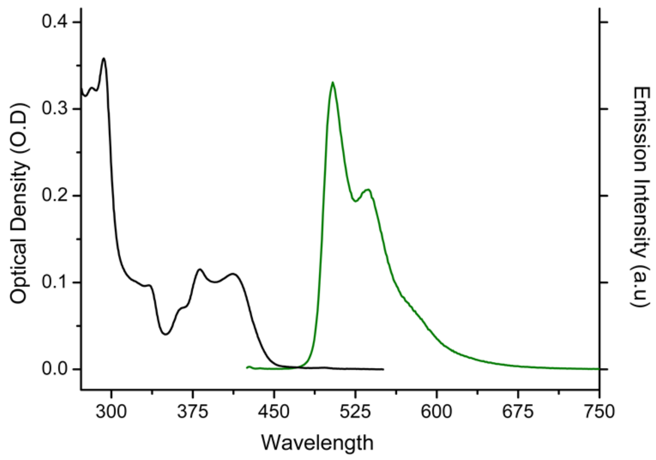

Figure S3: *Typical absorption (black) and emission (green) profile of Complex 1 in organic solvent (DMF).*

Figure S4: *Lack of Complex 1 induced DNA adducts*

1,2 (G,G) intrastrand DNA crosslinks were measured using monoclonal antibodies directed against these adducts [1]. Two hours following treatment cisplatin treated cell displayed significant levels of 1,2 (G,G) intrastrand crosslinks while complex 1 treated cells showed no greater level then untreated control cells.

(a) Measurement of 1,2 (G,G) Intrastrand-DNA adducts in genomic DNA cells following a two hour treatment with cisplatin or complex 1 at concentrations indicated. Error bars indicate SD, n=3. (b) Example dot blot used to generate data.

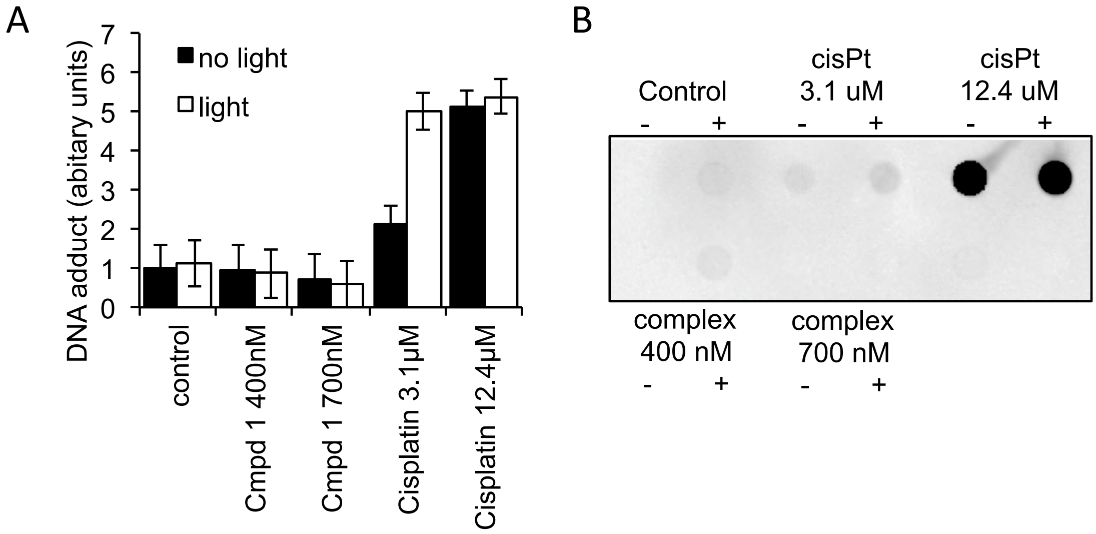

Supplementary Method: Measurement of 1,2 [GG] adducts in DNA

Cells were treated with complex 1 or cisplatin for 2 hours, harvested at relevant time points and frozen at -80°C until analysis. Genomic DNA was extracted using a DNeasy kit (Qiagen), treated with RNAse A (Sigma, UK) to remove damaged RNA. Exactly 1 µg DNA from each sample was loaded into a dotblotter (Biorad, CA), washed thoroughly with 20x SSPE buffer and baked (80°C, 30 mins). The

DNA was then blocked with 5% milk and probed using R-C18 monoclonal antibody (Oncolyse, Germany) raised against 1,2 [GG] intrastrand adducts [1] and then with an HRP-conjugated anti-rat IgG (#7707 – NEB, US). Dots were developed using a chemiluminescent substrate (GE Healthcare, UK), visualized and quantified using a LAS-3000 imager (Fujifilm).

1 Liedert, B., Pluim, D., Schellens, J. & Thomale, J. Adduct-specific monoclonal antibodies for the measurement of cisplatin-induced DNA lesions in individual cell nuclei. *Nucleic Acids Res* **34**, e47, doi:10.1093/nar/gkl051 (2006).
